# Oropharyngeal microbiome of a college population following a meningococcal disease outbreak

**DOI:** 10.1038/s41598-020-57450-8

**Published:** 2020-01-20

**Authors:** Adam C. Retchless, Cécilia B. Kretz, Lorraine D. Rodriguez-Rivera, Alexander Chen, Heidi M. Soeters, Melissa J. Whaley, Xin Wang

**Affiliations:** 1 Division of Bacterial Diseases, National Center for Immunization and Respiratory Diseases, Centers for Disease Control and Prevention, Atlanta, GA USA; 2 Present Address: Division of Scientific Education and Professional Development, Centers for Disease Control and Prevention, Atlanta, GA USA

**Keywords:** Metagenomics, Clinical microbiology, Microbiome

## Abstract

Asymptomatic oropharyngeal carriage of *Neisseria meningitidis* peaks in adolescence and young adulthood. Following a meningococcal disease outbreak at a U.S. college, we profiled the oropharyngeal microbiomes of 158 students to identify associations between bacterial community composition and meningococcal carriage or risk factors for carriage, including male gender, smoking, and frequent social mixing. Metagenomic shotgun sequencing identified 268 bacterial taxa at the genus or species level, with *Streptococcus, Veillonella*, and *Rothia* species being most abundant. Microbiome composition showed weak associations with meningococcal carriage and risk factors for carriage. *N. meningitidis* abundance was positively correlated with that of *Fusobacterium nucleatum*, consistent with hypothesized propionic acid cross-feeding. Additional species had positive abundance correlations with *N. meningitidis*, including *Aggregatibacter aphrophilus*, *Campylobacter rectus*, *Catonella morbi*, *Haemophilus haemolyticus*, and *Parvimonas micra*. *N. meningitidis* abundance was negatively correlated with unidentified *Veillonella* species. Several of these species are commonly found in dental plaque, while *N. meningitidis* is primarily found in the pharynx, suggesting that ecological interactions extend throughout the oral cavity. Although risk factors for meningococcal carriage do not strongly impact most bacterial species in the oropharynx, variation in the upper respiratory tract microbiome may create conditions that are more or less favorable for *N. meningitidis* carriage.

## Introduction

*Neisseria meningitidis* is a transient commensal of the human pharynx that also causes life-threatening diseases including meningitis and bacteremia^1^. Meningococcal carriage prevalence peaks in late adolescence and early adulthood^2^, corresponding to an increased risk of disease in this age group^1^. Risk factors for meningococcal carriage among adolescents and young adults in the United States include male gender, smoking, and frequent social mixing^3^. However, the influence of the pharyngeal microbiota on meningococcal carriage has been largely unexplored. A variety of bacterial species colonize the pharynx and other upper respiratory tract (URT) sites^4,5^, but there is currently little information about how they interact with *N. meningitidis* or how their populations are influenced by traditional risk factors for meningococcal carriage.

Understanding *N. meningitidis* ecology requires identification of microbial species that co-colonize the pharynx. Culture-based studies target particular species, often from the oropharynx, where *N. meningitidis* is most readily cultured^6^. These studies have provided evidence of competitive exclusion between *N. lactamica* and *N. meningitidis* in the oropharynx. This evidence includes the low prevalence of *N. lactamica* carriage among age groups with highest prevalence of *N. meningitidis* carriage^7,8^, and the reduced probability of isolating *N. meningitidis* from oropharyngeal swabs following inoculation with *N. lactamica*^9^.

Sequence-based, culture-independent approaches provide a more comprehensive assessment of the oropharyngeal bacterial community^10^, enabling both the discovery of unanticipated relationships and the evaluation of new hypotheses using previously collected data. For instance, *N. meningitidis* can utilize propionic acid as a carbon source, prompting the hypothesis that *N. meningitidis* growth is promoted by the presence of anaerobic bacteria that produce this short fatty acid by fermentation^11^. This cross-feeding hypothesis was initially evaluated with 16S rRNA gene data from bacteria in salivary specimens, identifying correlations between the proportional abundances of *Neisseria spp*. and two genera of anaerobic propionic acid producers, *Fusobacterium* and *Porphyromonas*. Further analysis of 16S rRNA genes showed that the average proportional abundance of both *Fusobacterium spp*. and *Porphyromonas spp*. increases through adolescence, in parallel to meningococcal carriage in the nasopharynx^11^.

Interpretation of 16S rRNA gene sequences can be limited by difficulty discriminating between *N. meningitidis* and other *Neisseria* species^12,13^. However, shotgun metagenomic sequencing can readily discriminate among the *Neisseria* species that are present in different parts of the oral cavity^14^. Furthermore, shotgun metagenomic sequencing enables identification of genes encoding metabolic enzymes in a bacterial community^15^.

To investigate how carriage of *N. meningitidis* among young adults may be influenced by variation in the bacterial community, we performed metagenomic shotgun sequencing on DNA obtained from oropharyngeal swabs collected from college students following a serogroup B meningococcal (MenB) disease outbreak in Providence, Rhode Island^3^. We evaluated the proportional abundances of each bacterial species in the oropharynx and identified species that were associated with meningococcal carriage or risk factors for meningococcal carriage.

## Results

### The oropharynx harbors a diverse bacterial community

Following four cross-sectional oropharyngeal carriage surveys of undergraduate students, 158 oropharyngeal swabs were selected for sequencing from a pool of 705 swabs collected during the third and fourth rounds from students who were between the age of 18 and 23 years, had not used antibiotics in the past 30 days, and had received both a quadrivalent meningococcal conjugate vaccine (MenACWY) and at least two doses of MenB-FHbp vaccine at least two weeks prior to swab collection. The 158 oropharyngeal swabs included 75 *N. meningitidis* culture-positive swabs (64 nongroupable, 6 serogroup E, and 5 serogroup B). The culture-positive and culture-negative swabs had similar composition with respect to the students’ carriage risk factors: gender, smoking, and frequent social mixing (Table 1; see methods for details).

**Table 1:** Characteristics of oropharyngeal swabs selected for shotgun metagenomic sequencing.

Swab characteristic				Swab counts	
*N. meningitidis* culture results	Smoking status^a^	Social mixing per week^b^	Gender	Sequenced (% of 158)	Available^c^ (% of 705)
Positive	Smoker	≥1	Male	12 (7.6%)	47 (6.7%)
Positive	Smoker	≥1	Female	14 (8.9%)	19 (2.7%)
Positive	Smoker	<1	Male	3 (1.9%)	3 (0.4%)
Positive	Smoker	<1	Female	1 (0.6%)	1 (0.1%)
Positive	Non-smoker	≥1	Male	10 (6.3%)	23 (3.3%)
Positive	Non-smoker	≥1	Female	14 (8.9%)	44 (6.2%)
Positive	Non-smoker	<1	Male	12 (7.6%)	12 (1.7%)
Positive	Non-smoker	<1	Female	9 (5.7%)	12 (1.7%)
Negative	Smoker	≥1	Male	12 (7.6%)	78 (11.1%)
Negative	Smoker	≥1	Female	11 (7.0%)	46 (6.5%)
Negative	Smoker	<1	Male	9 (5.7%)	17 (2.4%)
Negative	Smoker	<1	Female	9 (5.7%)	10 (1.4%)
Negative	Non-smoker	≥1	Male	9 (5.7%)	59 (8.4%)
Negative	Non-smoker	≥1	Female	11 (7.0%)	164 (23.3%)
Negative	Non-smoker	<1	Male	10 (6.3%)	51 (7.2%)
Negative	Non-smoker	<1	Female	12 (7.6%)	119 (16.9%)
**Total**				158 (100%)	705 (100%)

^a^Smoking in the past 30 days.

^b^Number of visits to bars, clubs, or parties per week.

^c^The swabs available for sequencing were limited to students who were between the age of 18 and 23 years, had received both a quadrivalent meningococcal conjugate vaccine (MenACWY) and at least two doses of MenB-FHbp vaccine at least two weeks prior to specimen collection, and had not used antibiotics in the past 30 days.

A total of 268 bacterial taxa were identified among the metagenomic sequences from the 158 oropharyngeal swabs; 239 of these taxa are species that are represented in the reference genome database, while 29 of the taxa are “unclassified” species belonging to different genera. The total proportional abundance of unclassified species ranges from 1.4% to 46.2% of the bacterial community (mean of 19.5%).

**Figure 1:**
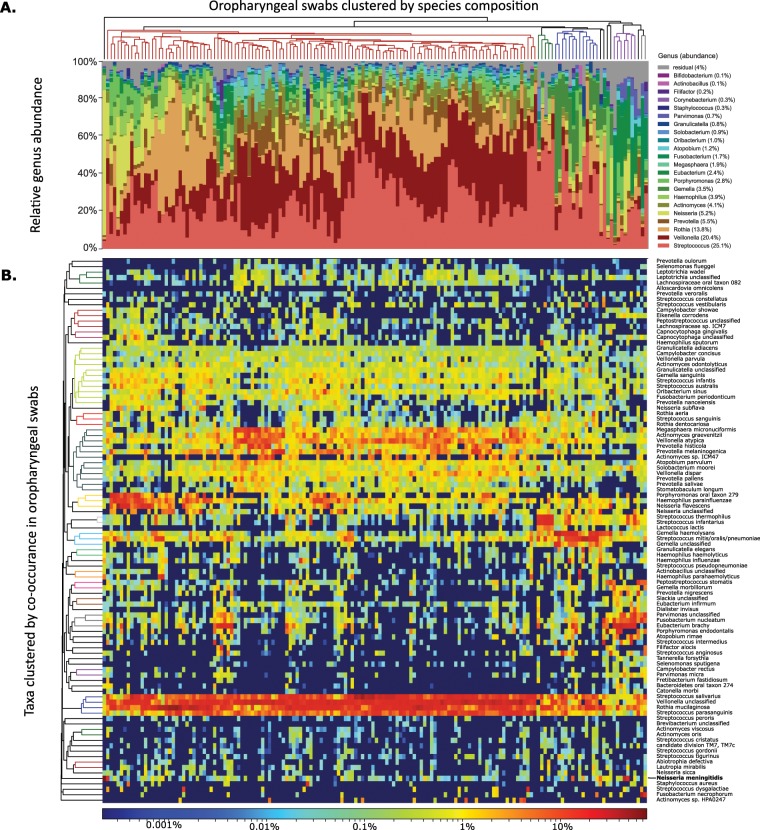
Bacterial species composition of 158 oropharyngeal swabs, clustered by species composition. (**A**) Abundance of each genus that accounts for over 10% of any swab. Average abundance is in parentheses in legend. (**B**) Heatmap showing the 100 most common bacterial taxa. Cells are color coded on a logarithmic scale to show the proportional abundance of each species (row) in each swab (column). In both panels, swabs are clustered according to average Bray-Curtis distances of their taxonomic profiles, represented by the dendrogram on the top. Taxa in panel B are clustered according to the Bray-Curtis distance of their abundance in each swab, shown by the dendrogram on the left. Colored portions of the dendrogram indicate groups for which distances are <70% of the maximum distance. *Neisseria meningitidis* is emphasized with bold font.

**Figure 2:**
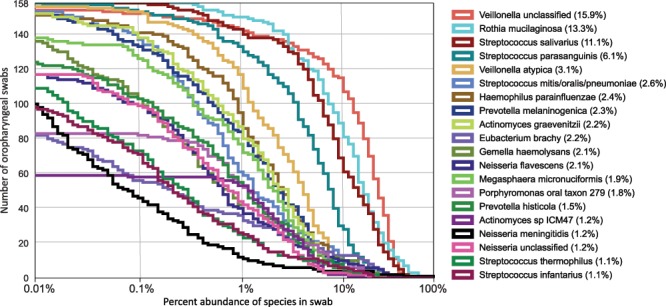
Abundance of dominant species among 158 oropharyngeal swabs. Curves show number of swabs in which each species was found at the given abundance; the y-intercept is equal to the number of swabs in which the species was identified. The twenty species shown have an abundance greater than 10% in one or more specimens, and a mean abundance greater than 1% overall (shown in parentheses in legend).

The proportional abundance of taxa varied substantially across specimens (Fig. 1). While five genera (*Streptococcus, Veillonella, Rothia¸ Prevotella*, and *Actinomyces*) were found in all 158 specimens, their proportional abundance varied by orders of magnitude between specimens (Fig. 1A). For instance, the most abundant genus, *Streptococcus*, ranged from 2.4% to 66%. Three species from two genera were detected in all 158 specimens: *Rothia mucilaginosa, Streptococcus salivarius*, and *Streptococcus parasanguinis* (Fig. 2). Clustering of swabs according to bacterial community composition placed 135 (85%) into a single group (Fig. 1, Supplemental Fig. 1). The effective number of taxa (exponential Shannon entropy) within the oropharyngeal specimens (13.4) is within the range of values calculated from taxonomic profiles of oral body sites generated by the Human Microbiome Project (HMP) using comparable methods (Supplemental Table 1). The taxonomic composition is most similar to that of the tongue dorsum (Supplemental Figs. 2 and 3; Mann-Whitney U, p < 0.001).

### Bacterial community composition is not associated with student traits

We tested whether the bacterial community differed between swabs based on *N. meningitidis* culture results, three carriage risk factors (the student’s gender, smoking behavior, and social mixing behavior), and two other potentially influential factors (the student having symptoms of URT infection in the prior two weeks, and the study round in which the swab was collected). The effective number of communities (exponential Shannon beta diversity) was no lower for pairs of swabs with the same values for any of these variables than for pairs of swabs with different values (Table 2, Supplemental Figs. 1 and 4). None of the six tests passed the 5% Bonferroni family-wise error rate, which required uncorrected p-values to be less than 0.83%.

**Table 2:** Median pairwise bacterial community diversity, within and between categories.

Characteristic	Categories, Value (N)		Median [Q1, Q3] of pairwise beta diversity^a^		p^e^
			Within the same category	Between categories	
*N. meningitidis* culture	Negative (83)	Positive (75)	1.320 [1.200, 1.499]	1.326 [1.203, 1.509]	0.08
Social mixing per week^b^	<1 (65)	≥1 (93)	1.326 [1.202, 1.515]	1.320 [1.201, 1.496]	0.93
Gender	Male (77)	Female (81)	1.319 [1.198, 1.500]	1.328 [1.205, 1.509]	0.02
Smoker^c^	No (87)	Yes (71)	1.318 [1.200, 1.501]	1.328 [1.204, 1.508]	0.05
URT infection^d^	No (119)	Yes (39)	1.326 [1.200, 1.512]	1.319 [1.204, 1.492]	0.91
Study round	Sep 2015 (78)	Mar 2016 (80)	1.323 [1.202, 1.506]	1.323 [1.201, 1.505]	0.55

^a^Effective number of communities based on Shannon entropy was measured for all 12,403 possible pairs of 158 swabs, with each pair assigned to “within” or “between” category depending on whether the two swabs were from the same or different category. The first and third quartiles are noted in brackets.

^b^Number of visits to bars, clubs, or parties per week.

^c^Smoking in the past 30 days.

^d^Symptoms of URT infection in past 2 weeks.

^e^Mann-Whitney U one-tailed p-value; a value < 0.0083 is required attain a Bonferroni-corrected significance of 0.05.

Likewise, only two significant correlations were identified between the proportional abundance of each taxon and variables describing the oropharyngeal specimen, using a multivariate linear model with a false discovery limit of 25%. The proportional abundance of *N. meningitidis* in the metagenomic data was significantly higher among swabs that were positive for *N. meningitidis* by culture (p = 3.4 × 10^−4^); the median proportional abundance was 3.57 × 10^−4^ in the Nm culture-negative swabs (99% CI, 1.72 × 10^−4^–9.65 × 10^−4^) and 1.48 × 10^−3^ in the Nm culture-positive swabs (99% CI, 4.92 × 10^−4^–3.98 × 10^−3^). *S. salivarius* was associated with females (p = 3.8 × 10^−4^); the median abundance was 6.0 × 10^−2^ among males (99% CI, 4.2 × 10^−2^–8.7 × 10^−2^) and 9.8 × 10^−2^ among females (99% CI, 7.4 × 10^−2^–1.7 × 10^−1^). No associations were identified with the remaining variables (study round, URT infections, smoking, or social mixing).

### ‘*N. meningitidis*’ abundance is correlated with the propionic acid producer, *Fusobacterium nucleatum*

Catanazzi *et al*. proposed that *N. meningitidis* growth in the oral cavity is promoted by the presence *Fusobacterium spp*. or *Porphyromonas spp*., which produce propionic acid by anaerobic fermentation^11^. Of the *Fusobacterium* or *Porphyromonas* species detected by metagenomic sequencing, only *F. nucleatum* had a significant compositional correlation with *N. meningitidis* (r = 0.22, p < 0.001). None of the remaining three *Fusobacterium* species (*F. gonidiaformans, F. necrophorum, F. periodonticum*) nor the four *Porphyromonas* taxa (*P. catoniae, P. endodontalis, P. gingivalis*, *Porphyromonas* oral taxon 279) had statistically significant compositional correlations with *N. meningitidis* (Supplemental Table 2).

Functional analysis of gene sequences in the metagenomic data indicated the potential for propionic acid production through the L-1,2-propanediol degradation pathway; no other pathway for fermentation to propionic acid was confidently identified. The L-1,2-propanediol degradation pathway was identified in 57 specimens; it was attributed to *F. nucleatum* in 18 specimens, to *F. periodonticum* in 27 specimens, and to both *F. nucleatum* and *F. periodonticum* in 12 specimens. The normalized abundance of genes for the L-1,2-propanediol degradation pathway was not correlated with the proportional abundance of *N. meningitidis* (Spearman r = −0.11, p = 0.16).

### ‘*N. meningitidis*’ abundance is correlated with other species

To identify potential interactions between *N. meningitidis* and any of the 267 taxa, we used three different correlation coefficients (Pearson’s, Spearman’s, and compositional) to test for correlations in proportional abundance between taxa. Six different taxa had significant positive correlations with *N. meningitidis* according to all three tests: *Aggregatibacter aphrophilus*, *Campylobacter rectus*, *Catonella morbi*, *Haemophilus aegyptius*, *Haemophilus haemolyticus*, and *Parvimonas micra* (Table 3). *Haemophilus aegyptius* is commonly described as a biogroup of *H. influenzae* rather than a separate species^16^; the Pearson correlation of *H. influenzae* and *N. meningitidis* abundances was not significant (r = 0.06; p = 0.46), but the Spearman (r = 0.34, p = 1.0 × 10^−5^) and compositional correlations (r = 0.17, p = 0.004) were significant. The only taxon for which all three tests identified a significant negative correlation with *N. meningitidis* was unclassified *Veillonella*, representing one or more *Veillonella spp*. that could not be identified at the species level. Notably, *N. lactamica* did not have a significant correlation with *N. meningitidis* (Supplemental Table 2).

**Table 3:** Bacterial taxa whose proportional abundance correlates to that of *N. meningitides*.

Taxon	Pearson *r*	(p)	Spearman *r*	(p)	SparCC	(p)
**Positive correlation**						
*Aggregatibacter aphrophilus*	0.22	(5.6 × 10^−3^)	0.17	(3.6 × 10^−2^)	0.10	(6 × 10^−3^)
*Campylobacter rectus*	0.31	(7.4 × 10^−5^)	0.27	(5.4 × 10^−4^)	0.15	(<1 × 10^−3^)
*Catonella morbi*	0.26	(8.4 × 10^−4^)	0.22	(4.8 × 10^−3^)	0.08	(7 × 10^−3^)
*Haemophilus aegyptius*	0.21	(9.7 × 10^−3^)	0.25	(1.7 × 10^−3^)	0.08	(3 × 10^−3^)
*Haemophilus haemolyticus*	0.20	(1.1 × 10^−2^)	0.37	(1.7 × 10^−6^)	0.17	(<1 × 10^−3^)
*Parvimonas micra*	0.29	(2.5 × 10^−4^)	0.25	(1.7 × 10^−3^)	0.16	(<1 × 10^−3^)
**Negative correlation**						
*Veillonella* unclassified	−0.22	(5.3 × 10^−3^)	−0.25	(1.7 × 10^−3^)	−0.35	(<1 × 10^−3^)

Note: p-values are not corrected for multiple hypothesis testing. Listed species had False Discovery Rate ≤25% for each of the three tests shown.

## Discussion

To better understand the ecology of *N. meningitidis*, we examined the oropharyngeal microbiome among young adults at a U.S. college where 20%–24% of surveyed students carried meningococcal bacteria, as detected via culture^3^. Previous DNA-based profiling of the oropharyngeal microbiome has used amplicon sequencing of 16S rRNA genes for genus-level analysis of bacterial communities^4,17,18^. The major oropharyngeal bacterial genera identified here among college students were similar to those described previously among adult populations such as United States active duty military personnel^17^ and a portion of the German National Cohort study^18^. The most prominent differences were a high abundance of *Rothia spp*. and a low abundance of *Leptotrichia spp*. among this college population. Shotgun metagenomic sequencing obtains species-level resolution for most of the bacterial community, but published shotgun metagenomic sequence data for the oropharyngeal microbiome is limited; comparison of these oropharyngeal communities’ profiles to the profiles published by the Human Microbiome Project showed that they were most similar to the communities of the tongue dorsum.

The overall bacterial community profile was not associated with any of the meningococcal carriage risk factors that we tested. Only one bacterial species was associated with any student trait tested here; *S. salivarius* was detected in all swabs but was more abundant among females. While we did not identify any association between smoking and bacterial taxon abundance, previous research has found that tobacco smoking is associated with differences in bacterial species abundance in oral rinse^19^ and nasopharyngeal^20^ specimens. These studies differed from our analysis of oropharyngeal specimens in several respects, including the examination of different body sites, older populations, and 16S rRNA gene sequencing rather than shotgun metagenomic sequencing. Furthermore, the oral rinse microbiome study of Bornigen *et al*.^19^ specified the intensity of cigarette use, finding that most of the differences in taxon abundance were primarily due to the comparison between regular cigarette smokers and cigarette non-smokers. In contrast, our smoking variable included regular cigarette smokers, occasional cigarette smokers (in the past 30 days), and marijuana smokers. The inclusion of a broader set of behaviors as “smoking” may have diminished the apparent association between smoking and bacterial taxon abundance.

Patterns of co-occurrence among microbial species can provide insights into ecological relationships, such as competition and mutualism^21^. Among the proposed ecological relationships of *N. meningitidis* is competition with *N. lactamica* in the pharynx, resulting in lower meningococcal carriage among individuals colonized with *N. lactamica*^9^. In this study, no significant association between *N. lactamica* and *N. meningitidis* abundances was identified. This is perhaps due to the low prevalence of *N. lactamica* carriage among older adolescents^7,8^, which was reflected in the detection of *N. lactamica* in only 18 of the 158 swabs. Ultimately, the metagenomic data indicate that competition with *N. lactamica* was not a substantial contributor to the variation in *N. meningitidis* carriage within this population.

*N. meningitidis* is capable of using propionic acid as a carbon source, and therefore may benefit from cross-feeding with propionic acid producers^11^. Studies based on 16S rRNA gene sequencing have shown that in saliva, the average proportional abundance of the anaerobic propionic acid producing genera *Fusobacterium* and *Porphyromonas* increases through adolescence, in parallel to meningococcal carriage in the nasopharynx^11^. The metagenomic data of the current study improved on prior 16S rRNA gene studies by estimating species-level abundances for all three genera, thereby identifying a significant positive correlation between *N. meningitidis* and *F. nucleatum*. The metagenomic data also confirmed the presence of *F. nucleatum* genes encoding a fermentation pathway that produces propionic acid; however, the abundance of genes for this pathway within the full microbial community was not correlated with *N. meningitidis* abundance.

Interactions between *F. nucleatum* and *N. meningitidis* may be limited by their tendency to grow in different locations within the URT – the gingival crevice for *F. nucleatum*^22^ and the pharynx for *N. meningitidis*. However, interactions may be mediated by saliva^23^, in which propionic acid levels can reach 3.3 mM^24^. The correlations in our metagenomic data suggest that *N. meningitidis* may interact with other taxa that are thought to grow in dental plaque and the gingival crevice, particularly *A. aphrophilus*, *C. rectus*, and *C. morbi*^25–28^; *H. haemolyticus* is also commonly found in dental plaque, as well as the pharynx^29^.

*Veillonella* species (for which a negative correlation with *N. meningitidis* was found) are commonly found in dental plaque, buccal mucosa, and the tongue^26^. Of note, like members of the genera *Fusobacterium* and *Porphyromonas*, members of *Veillonella* are anaerobic producers of propionic acid. While many *Veillonella* genomes encode genes for fermentation to propionic acid (e.g. *V. atypica*)^30^, these genes were not confidently identified in our metagenomic data. In contrast to members of *Fusobacterium* and *Porphyromonas*, members of *Veillonella* are abundant in the oral cavity throughout life^26^ and have been found to be more abundant among younger adolescents than older adolescents^31^.

The significant correlations identified between the proportional abundances of *N. meningitidis* and other bacterial species suggests possible beneficial or antagonistic interactions between these species and *N. meningitidis*. However, the bacterial species abundance is also affected by factors that were not examined in this study, such as oral and gastrointestinal health^19,32^. Such unmeasured and untested factors could influence the correlations between the abundances of *N. meningitidis* and other species. For instance, the negative correlation between *N. meningitidis* and *Veillonella* abundances could reflect the tendency for their abundances to change with age.

As sequencing technology and associated analysis tools advance, expanded investigation of URT ecology will be possible, providing context for studies of *N. meningitidis* transmission and host interactions. To understand meningococcal carriage and disease, further investigations will need to incorporate longitudinal observations of the microbiome as *N. meningitidis* carriage is gained or lost, particularly alongside medical interventions such as vaccination and antimicrobial treatments. Sampling from additional locations of the URT^5^ may help explain the correlations that we detected among the abundances of certain bacterial species that typically grow in dental plaque and the abundance of *N. meningitidis*. Examination of the nasopharynx is also important, since it is known to harbor *N. meningitidis*^6^, yet its bacterial community is dominated by different genera than the oropharynx (e.g. *Staphylococcus, Corynebacterium*, and *Moraxella*^17^). Further microbiome profiling can identify the large-scale structure of microbial communities, and guide more detailed studies to evaluate bacterial physiology and micron-scale interactions *in situ*. Ultimately, understanding the URT microbiome will improve knowledge for a variety of diseases, ranging from oral cancer^19^ to bacterial infections^4^.

## Methods

### Specimen collection

Oropharyngeal swabs were collected during four cross-sectional carriage surveys performed alongside a MenB-FHbp (factor H binding protein) vaccination campaign in response to the outbreak, as described by Soeters, *et al*.^3^. A total of 1,121 undergraduate students participated in the third and fourth study rounds (September 2015 and March 2016), during which one prong of a bifurcated swab was preserved for sequencing in 1 ml skim milk/tryptone/glucose/glycerin medium (STGG) on ice^33^, then frozen at −80 °C. The other prong was used for culture-based detection of *N. meningitidis* by plating on a modified Thayer-Martin (MTM) agar plate (BD BBL, Franklin Lakes, New Jersey) that was incubated at 37 °C with 5% CO_2_ and checked for growth at 24, 48, and 72 hours^3^. Colonies with typical *Neisseria* morphology were subcultured onto a blood agar plate (BD BBL), and *N. meningitidis* cultures were identified using Gram staining (BD BBL), oxidase testing (Hardy Diagnostics, Santa Maria, California), *sodC* rt-PCR, and API *Neisseria-Haemophilus* strip testing (bioMerieux, Durham, North Carolina)^34^. *N. meningitidis* isolates were serogrouped by slide agglutination^34^. Participants completed a short, self-administered questionnaire including potential meningococcal carriage risk factors, including gender, smoking (tobacco or marijuana in the past 30 days), frequent social mixing (visits to bars, clubs, or parties at least once per week), and recent URT infections (symptoms in the previous two weeks).

The carriage evaluation^3^ was approved by the Rhode Island Department of Health Institutional Review Board (IRB) (#IRB 2015 – 02) and the Providence College IRB (#15-062), and was determined to be public health non-research by the CDC National Center for Immunization and Respiratory Diseases (#2015 6436) and did not require CDC IRB approval. All participants provided written informed consent to participate in the carriage evaluation^3^ and for their oropharyngeal specimens to be stored for future research. This microbiome study used de-identified specimens and questionnaire data and was determined to not involve human subjects (#2016 6651) and did not require CDC IRB approval.

### DNA extraction and sequencing

DNA was extracted from 200 μL of STGG medium using QIAamp DNA Microbiome Kit (Qiagen) as per manufacturer protocol. DNA concentrations were measured using a Qubit 2.0 fluorometer with dsDNA High Sensitivity reagents (Invitrogen, Grand Island, NY, USA). The eluted DNA concentration ranged from 0.2 to 5 ng/μL.

Sequencing libraries were prepared from 2 ng of DNA using Nextera XT DNA library preparation kit following the manufacturer’s protocol (Illumina, San Diego, CA, USA). The resulting libraries were sequenced on three Rapid-Runs of an Illumina HiSeq 2500 (CDC Genomic Core facility) to obtain 250 bp paired-ends reads. Sixteen specimens with exceptionally low numbers of reads (<100,000 reads) were sequenced again on an Illumina MiSeq using a fresh library preparation.

### Metagenomic sequence analysis

Human DNA was removed by mapping reads to the hg38 reference genome using Bowtie2 (v2.2.9; local alignment mode)^35^, retaining a median of 94.8% (interquartile range: 85.7–98.4%) of reads with a minimum of 336,841 read pairs per specimen. Unmapped reads were submitted to SRA BioProject PRJNA428766. PCR duplicates were removed with bbtools-clumpify (v37.41)^36^, then Nextera adapter sequences and low-quality base calls were trimmed from reads with cutadapt (v1.8.3; options n = 5, trim-n = True, m = 50)^37^. The proportional abundance of bacterial species was estimated using MetaPhlAn2 (v2.2.0)^38^, which is a precise method for species detection (i.e. it rarely reports the presence of species that are not in a specimen)^39^. This precision is achieved by identifying marker genes that clearly distinguish different species, and if a sufficient number of these marker genes cannot be detected to confidently identify a species, MetaPhlAn2 conservatively limits its reporting to the genus or a group of species (e.g., *Streptococcus mitis/oralis/pneumoniae*). Data from the Human Microbiome Project (HMP) was retrieved as MetaPhlAn2 abundance files from the HMP website^40^.

Community taxonomic diversity of both the HMP specimens and the specimens from this study was calculated from MetaPhlAn2 taxonomic profiles using an exponential transformation of Shannon entropy to describe the effective number of taxa within a specimen (alpha) and the effective number of distinct communities in a collection of specimens (beta)^41^. Alpha diversity approaches a lower limit of 1 when a single species dominates the community; alpha diversity is equal to the number of observed taxa in a specimen when taxa are equally abundant in a specimen. Beta diversity approaches a lower limit of 1 when specimens have identical taxonomic profiles; beta diversity equals the number of specimens in the collection when there are no shared taxa between the specimens.

Associations of taxon abundance with specimen characteristics were calculated with MaAsLin (v0.0.4, default parameters)^42^, which applies a multivariate linear model to associate the abundance of each taxon with the available metadata. MaAsLin adjusts for multiple hypothesis testing by first applying a Bonferroni correction for each taxon’s association with each value of each metadata variable, then calculating a False Discovery Rate (FDR) over all taxa and metadata variables, reporting associations with FDR ≤ 25%. Metadata variables included in the multivariate linear model were: study round; *N. meningitidis* culture results; gender; smoking; social mixing; URT infection; and sequencing run.

Compositional correlations calculated with SparCC (v0.1)^43^ were used to assess correlated taxon abundances. Calculations were performed using pseudo-counts that were generated by multiplying proportional abundances by 1,000; this is a conservative scaling factor, as the proportional abundance table for each specimen was generated from at least 336,841 non-human read pairs and contained at least one taxon with proportional abundance under 0.001. Tests for previously predicted correlations between *N. meningitidis* and *Fusobacterium spp*. or *Porphyromonas spp*. were considered significant if the compositional correlation was more extreme than 99.375% of the correlation coefficients from permutated matrices (two-tailed, 1,000 permutations; Bonferroni adjustment for 8 tests with α ≤ 5%). When testing for associations that had not been previously hypothesized, a Benjamini-Hochberg FDR^44^ was applied to account for the large number of comparisons (267), and specificity was increased by using both Pearson and Spearman correlations to confirm associations (two-tailed t-test)^21^, controlling for FDR ≤ 25% for each test.

A functional interpretation of metagenome data was performed on each specimen using HUMAnN2 v2.9^15^ (default parameters, UniRef90 database). The potential for propionic acid production was assessed based on the presence of genes from each of the five pathways within the “Fermentation to Propanoate” class on the MetaCyc website^30^: “L-1,2-propanediol degradation pathway” (PWY-7013), “L-alanine fermentation to propanoate and acetate” (PROPFERM-PWY), “L-glutamate degradation VIII” (PWY-5088), and “pyruvate fermentation to propanoate” (P108-PWY and PWY-5494). The abundance of each pathway was measured at the community level and normalized to “copies per million” reads using the human2_renorm_table script. Confident detection of pathways required the coverage value to exceed 0.5.

Data analysis was performed with SciPy^45^ with scikit-bio version 0.5.2.

## Supplementary information

Supplementary information.

## Data Availability

The dataset supporting the conclusions of this article is available in the Short Read Archive repository, BioProject PRJNA428766.
