# Supplementary material for: Oropharyngeal microbiome of a college population following a meningococcal disease outbreak
Source: Sci Rep. 2020 Jan 20;10:632. doi: 10.1038/s41598-020-57450-8 (PMC6971049; doi:10.1038/s41598-020-57450-8)
Supplement: Supplementary file 1 — Supplementary information. [file 41598_2020_57450_MOESM1_ESM.pdf]

Supplemental Figures and Tables for: **Oropharyngeal microbiome of a college population following a meningococcal disease outbreak**

Authors: Adam C. Retchless, Cécilia B. Kretz, Lorraine D. Rodriguez-Rivera, Alexander Chen, Heidi M. Soeters, Melissa J. Whaley, Xin Wang

Supplemental Figure 1. Characteristics of each oropharyngeal specimen, clustered according to average Bray-Curtis distances.

Supplemental Figure 2. Principal coordinates analysis of community composition for oropharynx and body sites sampled by the Human Microbiome Project (HMP).

Supplemental Figure 3. Similarity of oropharyngeal bacterial community profiles to those reported by the HMP.

Supplemental Figure 4: Principal coordinates analysis of beta diversity among oropharyngeal specimens.

Supplemental Table 1. Species diversity within and between specimens taken from oropharynx and body sites sampled by the HMP.

Supplemental Table 2. Detection of *Porphyromonas spp.*, *Fusobacterium spp.,* and *N. lactamica* and correlations of their proportional abundances with that of *N. meningitidis*.

Oropharyngeal specimens clustered by species composition

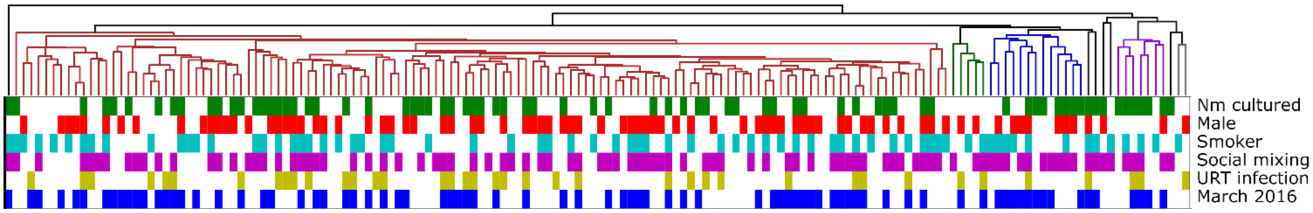

Supplemental Figure 1. Characteristics of each oropharyngeal specimen, clustered according to average Bray-Curtis distances, as in Figure 1. Colored cells represent: *N. meningitidis* cultured from the specimen (white: *N. meningitidis* not cultured); male (white: female); smoker (white: non-smoker); weekly social mixing (white: less frequent social mixing); upper respiratory tract (URT) infection (white: no recent URT infection); specimen collected in March 2016 (white: collected in September 2015). Colored portions of the dendrogram indicate groups for which distances are <70% of the maximum distance.

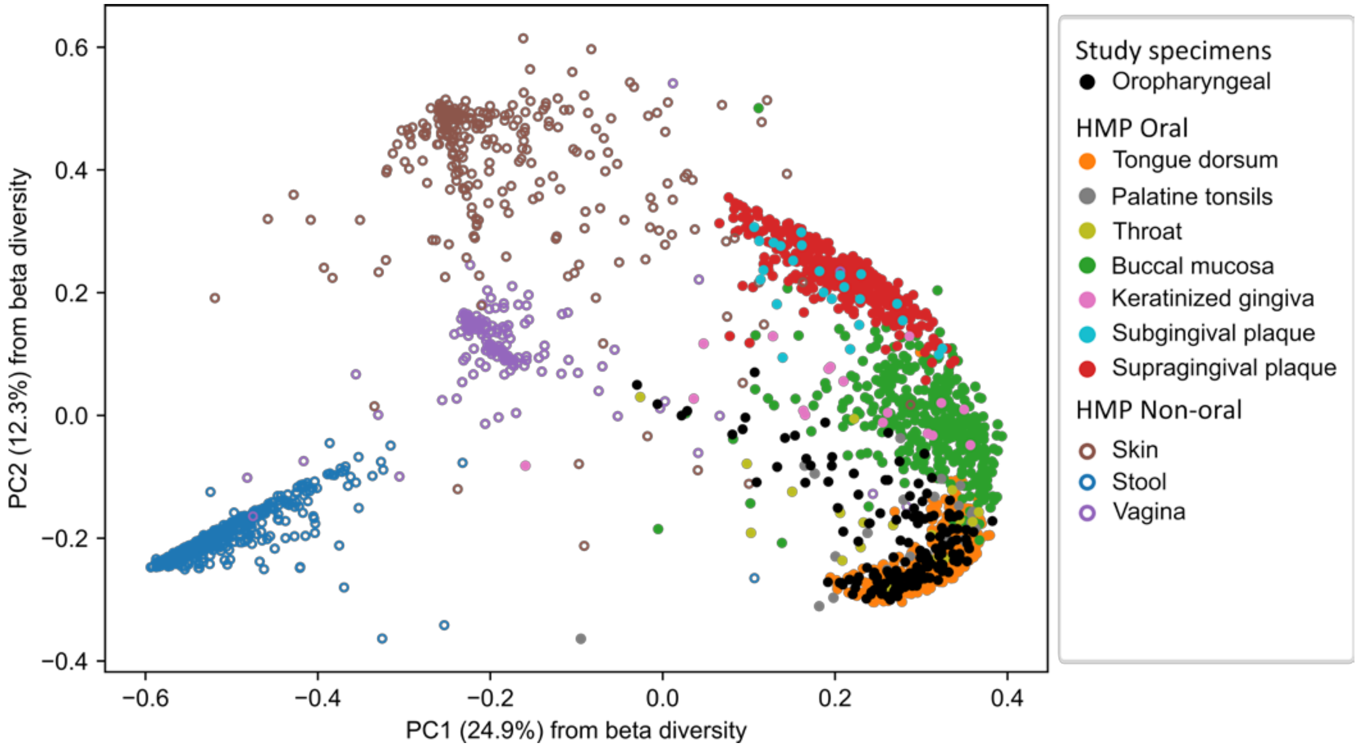

Supplemental Figure 2. Principal coordinates analysis of community composition of the oropharynx and body sites sampled by the Human Microbiome Project (HMP), based on pairwise beta diversity (exponential Shannon) values. Body sites with fewer than 10 specimens are not shown (hard palate, saliva).

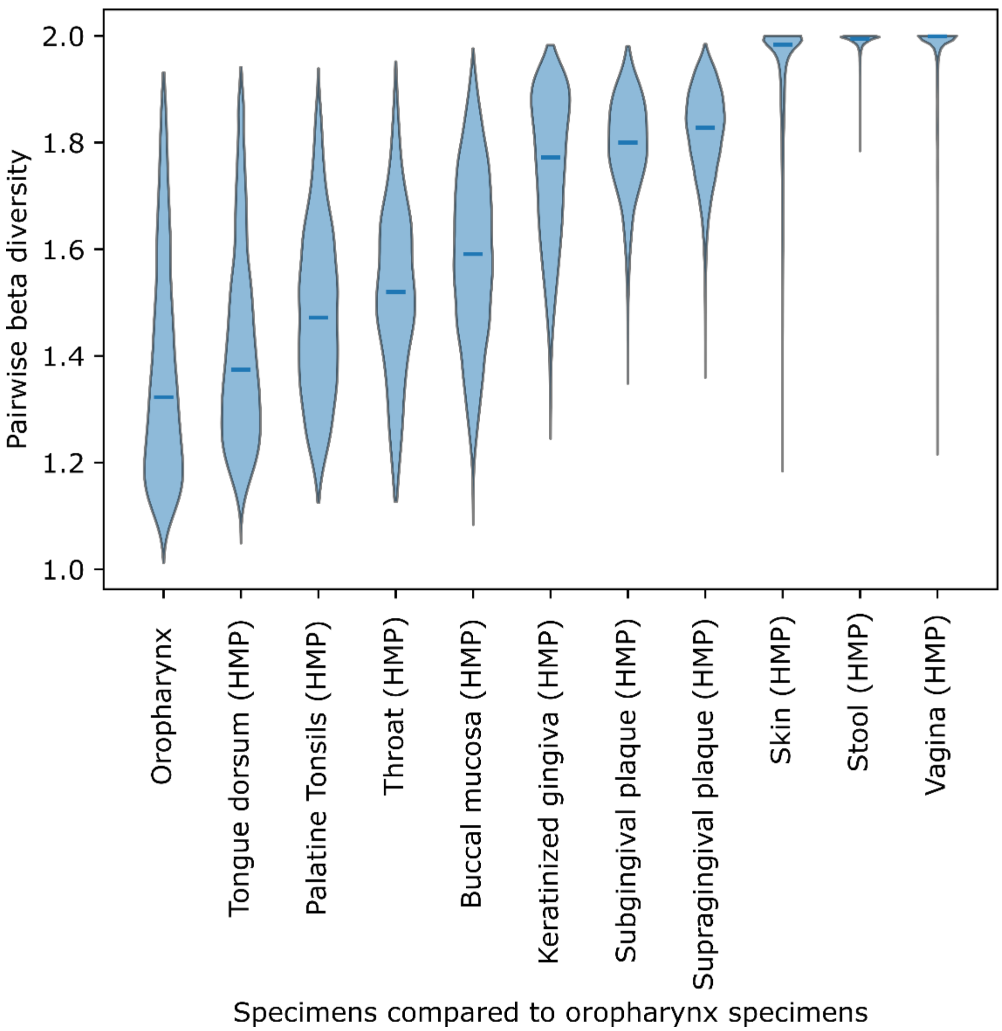

Supplemental Figure 3. Violin plot showing the distribution of pairwise beta diversity (exponential Shannon) values of specimens from the oropharynx compared to themselves or to specimens from body sites surveyed by the Human Microbiome Project (HMP). HMP body sites with fewer than 10 specimens are not shown (hard palate, saliva). The beta diversity is significantly lower for pairwise comparisons to the HMP tongue dorsum communities than for pairwise comparisons to any other HMP community (Mann-Whitney U test; $p < 0.001$ for each of 9 tests comparing values for tongue dorsum to values for each of the other HMP specimen sites)

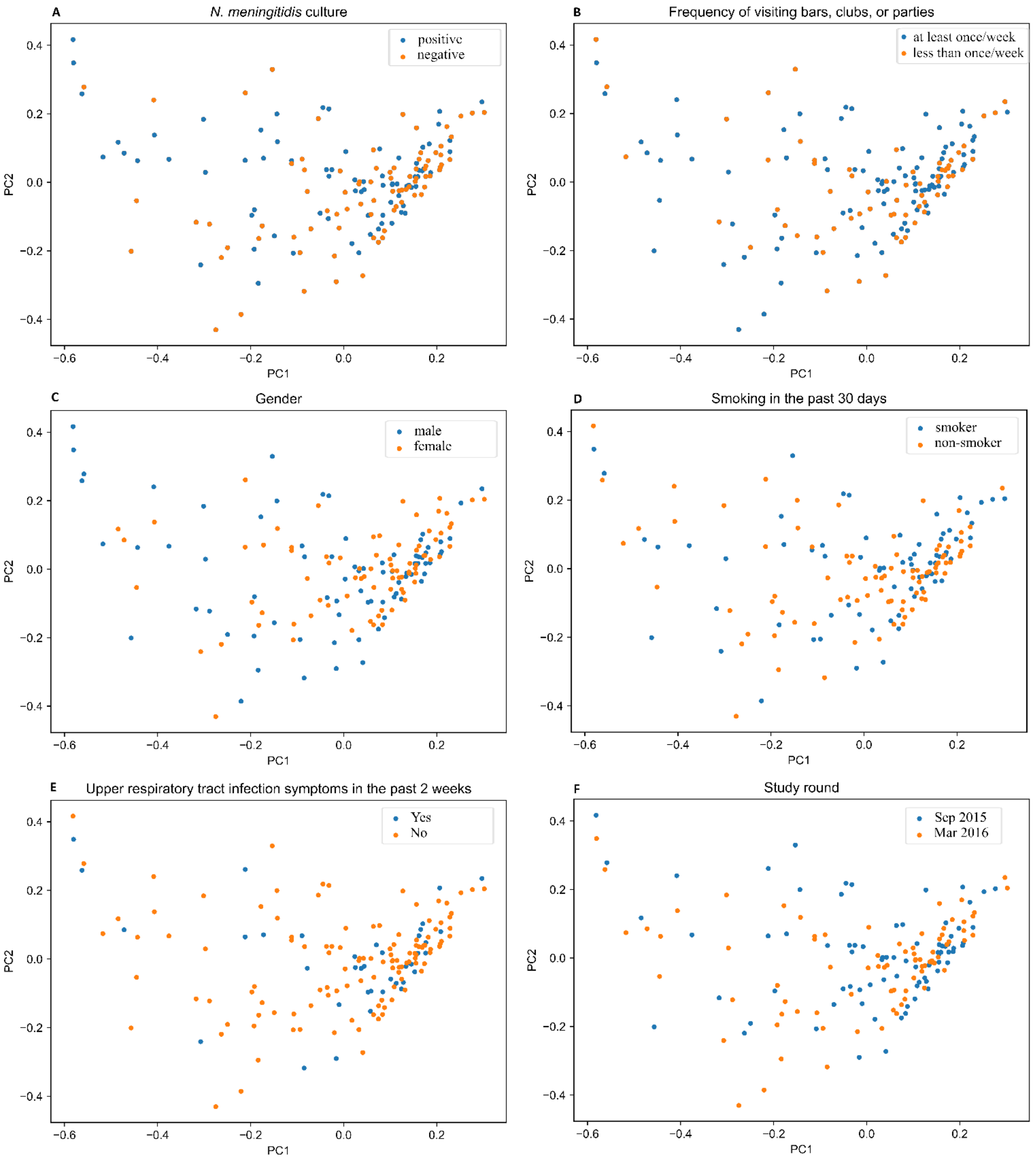

Supplemental Figure 4: Principal coordinates analysis of beta diversity (exponential Shannon) among oropharyngeal specimens. Each panel is color-coded according to a different trait, as described in Table 4: *N. meningitidis* culture (A); Social mixing (B); Gender (C); Smoking (D), Recent upper respiratory tract infection (E), and Study round (F)

Supplemental Table 1. Species diversity within and between specimens taken from each body site

Body site	Specimen count	Alpha diversity[a]	Pairwise beta diversity[b]
**Oropharynx**	158	13.4	1.323
**HMP Tongue Dorsum[c]**	210[d]	18.2	1.240
**HMP Palatine Tonsils**	23	21.6	1.425
**HMP Throat**	15	15.6	1.558
**HMP Buccal mucosa**	183	14.3	1.320
**HMP Keratinized gingiva**	13	10.0	1.471
**HMP Subgingival plaque**	20	27.5	1.374
**HMP Supragingival plaque**	191	21.2	1.312
**HMP Skin**	201	3.5	1.360
**HMP Stool**	249	12.3	1.521
**HMP Vagina**	118	1.7	1.901

a. Effective number of species per specimen taken from body site, exponential Shannon diversity.
b. Median effective number of communities between pairs of specimens from the same body site of different individuals. Possible values range from 1 to 2.
c. Microbiome composition obtained from Human Microbiome Project (HMP)
d. HMP data was limited to the first specimen taken from each individual for each body site

Supplemental Table 2. Detection of *Porphyromonas spp.*, *Fusobacterium spp.*, and *N. lactamica* and correlations of their proportional abundances with that of *N. meningitidis*.

**Taxon**	**Detection**[a]	**Detection with Nm**[b]	**Pearson *r***	**(p)**	**Spearman *r***	**(p)**	**SparCC**	**(p)**
Porphyromonas oral taxon 279	83	54	-0.06	(0.437)	-0.14	(0.086)	-0.10	(0.096)
Porphyromonas catoniae	11	10	-0.03	(0.743)	0.10	(0.234)	-0.01	(0.783)
Porphyromonas endodontalis	69	53	-0.02	(0.835)	0.19	(0.018)	0.08	(0.155)
Porphyromonas gingivalis	2	1	-0.01	(0.866)	0.01	(0.853)	0.00	(0.965)
Fusobacterium periodonticum	122	80	-0.07	(0.363)	-0.18	(0.025)	-0.08	(0.119)
Fusobacterium necrophorum	20	16	-0.02	(0.838)	0.18	(0.025)	0.07	(0.088)
Fusobacterium gonidiaformans	2	2	-0.01	(0.912)	0.16	(0.048)	0.01	(0.588)
Fusobacterium nucleatum	114	82	0.09	(0.283)	0.28	(<0.001)	0.22	(<0.001)
Neisseria lactamica	18	17	0.15	(0.062)	0.36	(<0.001)	0.03	(0.172)

a. Number of oral swabs where the proportional abundance of the species was greater than zero.

b. Number of oral swabs where the proportional abundance of both the listed species and *N. meningitidis* (Nm) was greater than zero.
